# Clinical and Radiographic Outcomes of Root Canal Obturation with Hydraulic Condensation and Tricalcium Silicate Bioceramic Sealer: A 12-Month Observational Study on Periapical Healing

**DOI:** 10.3390/jfb16110412

**Published:** 2025-11-05

**Authors:** Kostadin Zhekov, Vesela Stefanova

**Affiliations:** Department of Operative Dentistry and Endodontics, Faculty of Dental Medicine, Medical University of Plovdiv, 4000 Plovdiv, Bulgaria; vesela.stefanova@mu-plovdiv.bg

**Keywords:** BioRoot™ RCS, hydraulic condensation, PAI, periapical regeneration, tricalcium silicate bioceramic sealer

## Abstract

Successful endodontic treatment relies on effective shaping, disinfection and obturation. Calcium silicate sealers such as BioRoot™ RCS show promise due to their bioactivity and sealing properties, but more clinical evidence using standardized protocols is needed. This observational clinical study aimed to assess periapical healing at 6 and 12 months following single-visit root canal treatment using BioRoot™ RCS with hydraulic condensation in teeth with irreversible pulpitis or apical periodontitis. Sixty-six teeth were treated using a standardized protocol: ProTaper Gold instrumentation, sonic-activated irrigation, and hydraulic condensation with gutta-percha cone and BioRoot™ RCS. Periapical healing was evaluated using the periapical index (PAI) at baseline, 6 months, and 12 months. Clinical success was defined as functional, asymptomatic teeth and a PAI ≤ 2. Statistical analysis included repeated measures of ANOVA and McNemar’s test. All 66 teeth remained asymptomatic and functional of 12 months, yielding a 100% survival rate. Clinical success was confirmed in 97% of cases. PAI scores decreased significantly over time (*p* < 0.001) in apical periodontitis cases. Single-visit endodontic treatment with BioRoot™ RCS and hydraulic condensation demonstrated excellent clinical and radiographic outcomes. This approach promotes resolution of apical periodontitis in non-vital cases and supports the preservation of periapical health in teeth initially diagnosed with irreversible pulpitis.

## 1. Introduction

The success of endodontic treatment critically depends on the effective shaping, disinfection and three-dimensional sealing of the root canal system to prevent reinfection and promote periapical healing [1]. Over the past few decades, bioceramic materials have emerged as a promising alternative to traditional sealers, offering enhanced bioactivity, biocompatibility, and sealing ability [2,3]. Among these, tricalcium silicate bioceramic endodontic sealers such as BioRoot™ RCS (Septodont, France) have gained considerable attention due to their hydraulic setting properties, excellent adhesion to dentin, and potential to stimulate hard tissue regeneration [4,5,6].

Conventional endodontic sealers, such as zinc oxide-eugenol-based or epoxy resin-based materials, have been widely used and extensively studied. However, their shortcomings include potential cytotoxicity, dimensional instability, and lack of bioactivity, which have been associated with less favorable biological responses and may compromise the sealing ability over time [7]. Calcium hydroxide-based sealers, though biologically favorable, often suffer from high solubility and rapid dissolution, compromising the integrity of the root canal obturation. These limitations have driven the search for materials that not only provide hermetic sealing but also actively participate in the healing process [8,9].

Tricalcium silicate bioceramic sealers represent a novel class of hydraulic cements primarily composed of calcium silicates and related compounds. They set in the presence of moisture, enabling the sealer to cure even in the inherently wet environment of the root canal system. Their chemical composition endows them with bioactive properties, such as the release of calcium ions and the ability to induce hydroxyapatite formation at the dentin—sealer interface. These features are believed to contribute to enhanced sealing, reduced microleakage, and stimulation of periapical tissue repair [4,10,11,12].

The hydraulic condensation technique, which involves the use of bioceramic sealers in conjunction with a single gutta-percha cone, is increasingly employed in clinical practice [13]. This approach offers several practical advantages, including simplicity, reduced treatment time, and the potential for effective sealing when appropriate moisture control is ensured, although some in vitro studies have reported a greater incidence of voids compared to warm vertical compaction techniques [14]. Moreover, hydraulic condensation leverages the flow and setting characteristics of bioceramic sealers to fill accessory canals, dentinal tubules, and irregularities without additional compaction force [3,15].

BioRoot™ RCS is a tricalcium silicate-based root canal sealer supplied in powder and liquid form, requiring chairside manual mixing before application [8]. The material’s crystalline structure primarily consists of hydration products derived from its core components. Upon setting, the material forms calcium silicate hydrate (C-S-H) gel as the primary amorphous phase, which provides mechanical strength and sealing properties. Crystalline calcium hydroxide (portlandite) develops as a byproduct, contributing to the material’s alkaline pH and antimicrobial activity while releasing calcium ions for bioactivity. Over time, these ions interact with phosphate in tissue fluids to form crystalline hydroxyapatite on the sealer surface, enhancing biocompatibility and bonding to dentin. The sealer also contains zirconium oxide crystals that remain chemically stable as a radiopacifier, maintaining their monoclinic or tetragonal structure throughout the setting process without participating in hydration reactions. This combination of amorphous C-S-H gel and crystalline phases (calcium hydroxide, hydroxyapatite, and zirconium oxide) gives BioRoot™ RCS its distinctive bioactive properties and dimensional stability. The material’s unique composition promotes tissue regeneration while ensuring optimal sealing and antimicrobial action through its carefully balanced crystalline and amorphous components. Unlike some endodontic sealers, BioRoot™ RCS contains zirconium oxide as a radiopacifier, avoiding bismuth oxide, which has been linked to discoloration and is a subject of debate regarding biocompatibility concerns. Recent studies have raised issues regarding the long-term systemic safety of bismuth-containing materials, prompting the development of newer bioceramic sealers without this additive [4,7,8,10,11,12].

Clinical studies evaluating the performance of BioRoot™ RCS and other bioceramic sealers have demonstrated promising periapical regeneration results. Research by Gadzhula (2017) and Bardini et al. (2021) reported high survival and success rates in teeth with apical periodontitis treated with hydraulic condensation and BioRoot™ RCS, outperforming traditional sealers in comparable clinical settings [16,17]. Similarly, Zavattini et al. (2020) highlighted favorable healing assessed with advanced imaging modalities, including cone-beam computed tomography (CBCT) [6]. Nonetheless, some long-term studies and reviews, such as that by Seog et al. and a comprehensive review by Sfeir et al.’s study, have reported cases of comparable or variable long-term outcomes depending on sealer formulation stability and application protocols, underscoring the importance of both material selection and clinical technique [18,19].

Despite these encouraging outcomes, the available data are often limited by small sample sizes, heterogeneity in methodology, and varying follow-up periods and methods for healing assessment [6,16,17]. There are several well-designed clinical studies, published recently, that validate the long-term efficacy of calcium silicate-based sealers. Simon et al. (2025) and Hu et al. (2023) presented two years follow up results, while Spinelli et al. (2024) and Bardini et al. (2025) reported three and four years follow-ups, respectively [20,21,22,23]. Additionally, the influence of factors such as canal drying methods, irrigation activation techniques, and single-versus multiple-visit treatment regimens on the biological response warrants further investigation. Building upon this foundation, the present study introduces a novel perspective by investigating the healing process following hydraulic condensation with a bioceramic biomaterial under a rigorously standardized and controlled clinical protocol. All treatments were performed following identical operative parameters for instrumentation, moisture control, sealer placement, and obturation pressure, thereby eliminating procedural variability as a confounding factor. To our knowledge, such a degree of methodological standardization has not yet been reported in prospective in vivo research on endodontic biomaterials and obturation techniques.

Our study aims to evaluate the healing outcomes following single-visit endodontic treatment of irreversible pulpitis and apical periodontitis using the hydraulic condensation technique with BioRoot™ RCS sealer. We also seek to provide a comprehensive analysis of clinical and radiographic success at 6 and 12 months. The tested null hypothesis is that this technique shows significant improvement in radiographic PAI scores at 12 months.

## 2. Materials and Methods

### 2.1. Study Design and Patient Selection

This exploratory observational clinical study was conducted to evaluate the healing outcomes of root canal treatment using the hydraulic condensation technique with BioRoot™ RCS bioceramic sealer. A total of 66 teeth from patients requiring endodontic treatment were enrolled and followed for up to 12 months post obturation, in accordance with the quality guidelines for endodontic treatment recommended by the European Society of Endodontology [24]. The study protocol was approved by the institutional ethics committee (2021_RKNE_078C212D87f) and registered at ClinicalTrials.gov (Identifier: NCT07075354), and all patients provided informed consent prior to inclusion.

The inclusion criteria encompassed permanent teeth in patients aged over 18 years of both biological genders who were in good general health, diagnosed with irreversible pulpitis or apical periodontitis and thus requiring primary root canal treatment [25]. No retreatment cases were included. Both maxillary and mandibular single-rooted and multi-rooted teeth were included to represent a typical clinical spectrum. The clinical protocol—including anesthesia, rubber dam isolation, access cavity preparation, working length determination, instrumentation, irrigation, and obturation—followed the ESE 2006 recommendations for standardized endodontic treatment procedures and outcome assessment [24]. Due to the limited sample size, no separate subgroup analyses based on pulp diagnosis or tooth type were performed; all cases were analyzed together as a single cohort. Teeth with open apices (immature teeth), extensive resorption, or severe periodontal disease were excluded.

The required sample size was estimated based on published study by Bardini et al. reporting healing success rates of approximately 90% following root canal obturation using BioRoot™ RCS [23]. To detect a clinically relevant difference of at least 15% in success rates with a statistical power of 80% (β = 0.20) and a significance level of 5% (α = 0.05), a minimum of 60 teeth was required. To compensate for potential dropouts or protocol deviations, a total of 66 teeth were included in the final analysis. The sample size calculation was performed using G*Power software, version 3.1 (Heinrich Heine University, Düsseldorf, Germany).

A total of 102 teeth fulfilled the inclusion criteria. In total, 36 teeth were subsequently excluded as consent was withdrawn (25 teeth) or because upon commencing treatment one or more canals were deemed unnegotiable (11 teeth).

**Figure 1:**
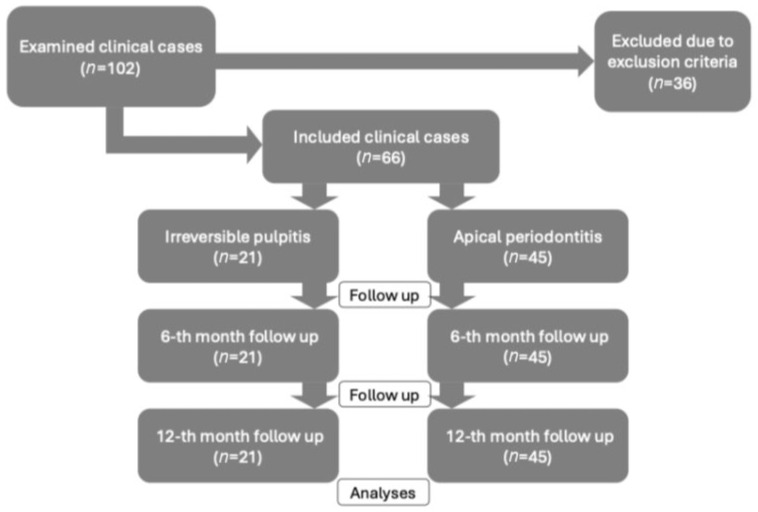
Flow diagram of the study recruitment, follow-up and analyses.

The design of the clinical study and patient flow is presented in Figure 1, in accordance with the CONSORT reporting guidelines for cohort studies.

### 2.2. Clinical Procedure

All treatments were performed by a single experienced endodontist using a standardized protocol. Following local anesthesia and rubber dam isolation, access cavities were prepared. Working length was determined using an electronic apex locator (Raypex 6, VDW GmbH, Munich, Germany) and confirmed radiographically.

The root canals were prepared using rotary NiTi instruments ProTaper Gold^®^ (PTG) (Dentsply Sirona, Wien, Austria) according to a crown-down approach, ensuring adequate apical enlargement and taper. Irrigation was performed with 2.5% sodium hypochlorite (NaOCl) throughout instrumentation, with approximately 2–3 mL delivered after each file using a side-vented 30G needle (NaviTip, Ultradent, Cologne, Germany). Following completion of instrumentation, the canals received a final rinse of approximately 5 mL NaOCl, activated with sonic energy via polymer tips to enhance debris removal and disinfection. A chelating solution, 17% Ethylenediaminetetraacetate (EDTA), was applied for 1 minute to remove the smear layer, followed by a final rinse of approximately 5 mL sterile saline to remove residual irrigants [26].

All cases were treated in a single visit. After the final irrigation sequence, the irrigant was removed from the canals by gentle aspiration with a syringe and side-vented needle. Each canal was then dried with a single calibrated sterile paper point, inserted until it reached working length and removed once it appeared moist but not wet. This ensured that the canal environment remained slightly moist, which is optimal for the hydration and setting of BioRoot™ RCS, while avoiding overdrying.

### 2.3. Obturation Technique

The hydraulic condensation technique was employed for root canal obturation. First the sealer was prepared according to the manufacturer’s instructions, involving manual mixing to ensure homogeneity and optimal handling properties. Second, the sealer was precisely delivered and distributed in the canal using an appropriate size Lentulo spiral, mounted on a contra angle handpiece and rotation speed of 600 rpm. Third a single standardized gutta-percha cone corresponding to the prepared canal size, coated with BioRoot™ RCS bioceramic sealer, was gently inserted into the canal to the working length. A heat carrier was used to cut the gutta-percha cone at the level of the orifice for each root canal. X-ray control of the root canal obturation was executed just after temporary coronal restoration. The type of the final restorations depended on the extent of hard dental tissue loss—direct composite restorations, indirect composite restorations and full coverage crowns. Teeth were definitively restored within 1 week under rubber dam isolation by the same operator. A self-etching dentinal bonding agent (Clearfil SE BOND, Kuraray, Osaka, Japan) was applied, light-cured for 30 s and layered with flowable (Premise Flowable, 3M ESPE, St. Paul, MN, USA) and composite (Filtek Z550, 3M ESPE, St. Paul, MN, USA) resins applied incrementally with 1.5 mm layers. Contemporary dental laboratory materials were used to produce indirect composite restorations and full coverage crowns. Patients were scheduled for follow-up visits at 6 and 12 months.

### 2.4. Clinical and Radiographic Evaluation

Clinical success was defined by the absence of symptoms (pain, swelling, or tenderness) and functional tooth preservation without mobility or other complications and a PAI ≤ 2. Radiographic evaluation was conducted using standardized two-dimensional digital periapical radiographs taken with a parallel technique at 6 and 12 months.

The periapical status was assessed using the periapical index (PAI), scoring lesions from 1 (healthy) to 5 (severe periodontitis with exacerbation). PAI-1 and PAI-2 scores were considered indicative of healed or healing sites, while scores of PAI-3 and above denoted persistent periapical pathology [27].

Digital periapical radiographs (Planmeca ProSensor HD) were taken at baseline, the 6-th month and the 12-th month, using a parallel technique and evaluated on calibrated 27-inch high-resolution monitors under standardized conditions. Two trained and calibrated examiners assigned a PAI score to each radiograph; in the case of a disagreement, the highest of the two scores was retained. Both examiners were board-certified endodontists with more than 10 years of experience. Calibration was performed on a sample of 20 radiographs not included in this study, with consensus discussions to resolve discrepancies. Inter-examiner reliability was assessed using Cohen’s kappa coefficient, which yielded a value of κ = 0.81, indicating almost perfect agreement according to Landis and Koch’s criteria. In multi-rooted teeth, the root with the highest score was used as the reference.

### 2.5. Statistical Analysis 

Statistical analyses were performed using statistical software (SPSS v.26, Inc., Chicago, IL, USA). Longitudinal changes in PAI scores were analyzed using repeated measures ANOVA with Bonferroni post hoc tests for pairwise comparisons between baseline, 6-month, and 12-month timepoints. Non-parametric Wilcoxon signed-rank tests complemented ANOVA for non-normally distributed data. Effect sizes were calculated using Cohen’s d. Categorical improvements in PAI scores were assessed with McNemar’s test. Data are presented as mean ± standard deviation for continuous variables (PAI scores). Statistical significance was set at *p* < 0.05.

## 3. Results

Sixty-six clinical cases of irreversible pulpitis (*n* = 21) and apical periodontitis (*n* = 45) were treated in this study. The patient cohort mean age was 41.83 ± 12.5 years, with the gender distribution showing a higher proportion of females—55% (*n* = 36), while males accounted for 45% (*n* = 30). Of the 66 treated teeth, 7 were anterior, 20 premolars, and 39 molars; 18 teeth were single-rooted and 48 were multi-rooted. All cases were followed up at 6 and 12 months, and the obtained data were statistically analyzed (Figure 1). At the 12-month follow-up, the success rate was 100% for irreversible pulpitis (21/21) and 97% for apical periodontitis (43/45), reflecting slightly more favorable outcomes in vital pulp cases.

**Figure 2:**
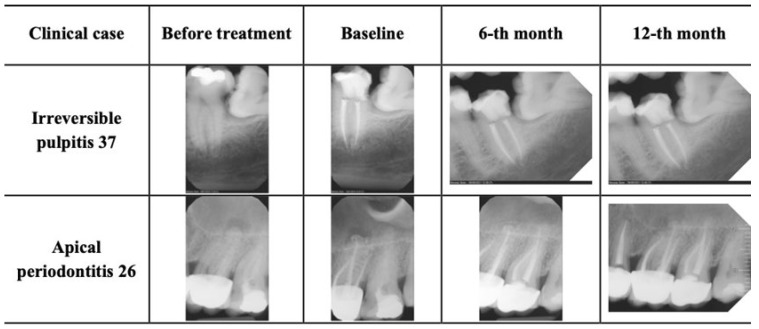
Representative radiographs of clinical cases.

All patients were monitored postoperatively through recall visits and follow-up contact. No flare-ups requiring additional treatment were recorded. Transient postoperative discomfort occurred in approximately 3% of cases, typically resolving within 2–3 days without intervention. At both the 6- and 12-month recalls, all treated teeth were functional and symptom-free. Coronal restorations remained adequate, with no signs of fractures or secondary caries (Figure 2).

**Figure 3:**
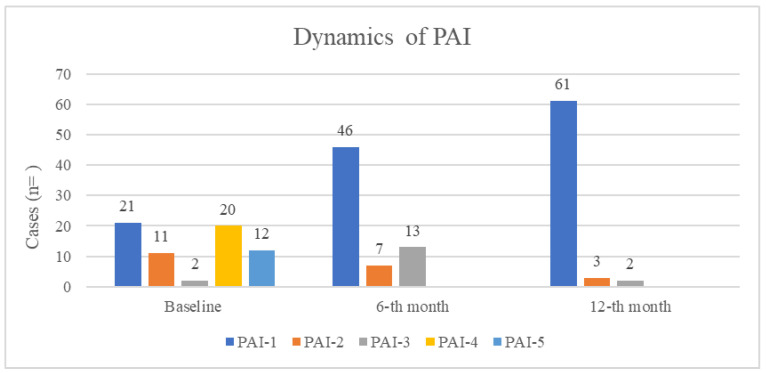
Dynamics of PAI on the 6-th month and on the 12-th month.

For the baseline PAI distribution immediately after endodontic treatment completion, radiographic assessment showed healthy periapical tissues (PAI-1) in 32% of cases (*n* = 21), while the remaining cases exhibited varying degrees of radiolucency: mild changes (PAI-2) in 17% (*n* = 11), moderate inflammation (PAI-3) in 3% (*n* = 2), and established or severe apical periodontitis (PAI-4 and PAI-5) in 30% (*n* = 20) and 18% (*n* = 12) of cases, respectively (Figure 3).

Follow-up examinations demonstrated progressive healing over time (Figure 3). At the 6-month evaluation, a marked improvement was observed, with PAI-1 cases increasing to 70% (*n* = 46) and PAI-2 cases decreasing to 11% (*n* = 7). The slight increase in PAI-3 cases (20%, *n* = 13) likely represented transitional healing stages, while a decrease in more severe radiolucency (PAI-4 and PAI-5) was already evident at this stage. By the 12-month follow-up, healing progression continued, with PAI-1 cases reaching 92% (*n* = 61) and residual mild changes (PAI-2 and PAI-3) present in only 5% (*n* = 3) and 3% (*n* = 2) of cases, respectively (Figure 3).

The overall survival rate was 100%, with all cases showing either complete resolution (92%) or continued improvement (8%) in the 12-th month. Notably, cases initially diagnosed with irreversible pulpitis (*n* = 21) maintained PAI-1 status throughout the study period, demonstrating no periapical pathology development. Among apical periodontitis cases (*n* = 45), the healing patterns were particularly noteworthy. Cases initially presented with severe radiolucency (PAI-5, *n* = 12) showed substantial improvement, with 42% achieving complete healing (PAI-1) by 12 months, while others demonstrated progressive reduction in lesion severity. Similarly, cases beginning with PAI-4 (*n* = 20) showed excellent outcomes, with 90% reaching PAI-1 status by final follow-up. Even cases with initially mild pathology (PAI-2, *n* = 11) showed near-complete resolution, with 91% progressing to PAI-1 (Figure 3).

Statistical analysis confirmed these clinical and radiographic observations. The calculated mean PAI scores were as follows: baseline = 2.86 ± 1.45; 6-th month = 1.50 ± 0.89 and 12-th month 1.11 ± 0.39. Repeated measures ANOVA demonstrated highly significant improvement in PAI scores over time (*p* < 0.001). The most dramatic change occurred between baseline and 6 months (*p* < 0.001, Wilcoxon signed-rank test), with effect size according to Cohen’s d = 1.65, followed by continued improvement to 12 months with further reduction to 1.11 ± 0.39 (*p* < 0.001). Pairwise comparisons using Bonferroni correction revealed significant differences between all timepoints (*p* < 0.003 for all comparisons) (Table 1).

McNemar’s test confirmed significant increases in PAI-1 prevalence at both follow-up intervals compared to baseline (*p* < 0.001). These results demonstrate not only the effectiveness of the treatment protocol but also the dynamic nature of periapical healing, with most improvement occurring in the first six months followed by more gradual refinement thereafter. Success rate comparisons show baseline: 32% PAI-1, 48% PAI-4/5 (pathology); 6-month: 70% PAI-1 (*p* < 0.001, McNemar’s test); 12-month: 92% PAI-1 (*p* < 0.001 vs. baseline and 6-month).

The complete absence of PAI-4/5 cases at follow-up and the high rate of PAI-1 achievement (92%) at 12 months provide compelling evidence for the treatment’s success in achieving periapical health.

## 4. Discussion

All 66 treated teeth remained functional and asymptomatic throughout the 12-month period, reflecting a complete survival rate. This supports the reliability of the hydraulic condensation technique and confirms that the use of BioRoot™ RCS results in radiographic success as well as sustained tooth survival and functional integrity over time. The lack of postoperative symptoms, swelling, or percussion sensitivity in any of the treated cases provides strong clinical confirmation of healing. Moreover, the 97% clinical success on the 12-th month, factoring in radiographic evolution to a PAI ≤ 2, aligns with or exceeds the outcomes reported in comparable studies [6,16,17].

The use of PAI scoring provided an objective, reproducible, and quantifiable approach for assessing healing, which is often lacking in prior clinical studies in which descriptors such as “normal,” “improved,” or “unsatisfactory” are used without formal scoring systems [27]. Our study included permanent teeth of all types. For multi-rooted teeth, the root demonstrating the most severe pathological involvement (i.e., the root with the highest PAI score) was selected as the reference for evaluation and treatment outcomes. This approach ensured consistent assessment across all tooth types while maintaining clinical relevance to the endodontic condition being treated. This methodological rigor further strengthens the validity of our outcomes and their comparability across future studies.

A strength of the present study lies in the systematic use of the PAI scoring system to evaluate periapical healing over time. The dynamic reduction in PAI scores across 6- and 12-month follow-ups clearly demonstrated tissue recovery and bone regeneration. At baseline, 51.5% of treated teeth (34/66) exhibited periapical radiolucency (PAI ≥ 3). By 12 months, 94.1% of these cases (32/34) had PAI scores reduced to ≤2, indicating successful healing. The remaining case demonstrated continued PAI improvement, though not yet fully resolved. Notably, teeth without baseline periapical radiolucency maintained PAI-1 throughout, underscoring the preventive capacity of the obturation protocol. Chemo-mechanical preparation is the cornerstone of successful endodontic treatment, and we acknowledge that effective cleaning and shaping, in conjunction with proper irrigation protocols, play a critical role in achieving periapical healing. In our study, standardized instrumentation and irrigation protocols were rigorously followed across all cases to ensure consistency and to minimize variability.

The outcomes of this study align to those reported in the literature. Bardini et al., in a prospective study of 39 teeth using hydraulic condensation with BioRoot™ RCS, reported 12-month healing rates of 76.92% (no periapical lesion) and 67.86% (with lesion). The clinical success reported in our study cannot be directly compared to the periapical healing rate described by Bardini et al. In their study, the primary outcome was strictly defined as: “Periapical healing”, including clinical and radiographic evidence of the healing of each tooth or the absence of apical periodontitis. Treatment success was defined according to strict criteria as the absence of pain or clinical evidence of inflammation or swelling and by conventional radiographic measures of complete healing/continuous presence of a normal periodontal ligament space (PAI score < 2).” In contrast, our definition of clinical success includes cases with PAI score 2, which—according to Bardini et al.—are classified as not healed under their stricter criteria. Their use of a less defined irrigation and drying protocol may have contributed to the lower success rates. Their control group with zinc oxide-eugenol sealer yielded even lower results, reinforcing the clinical value of bioceramics [17].

Gadzhula et al. observed a 100% healing rate at 12 months in a smaller cohort of 11 teeth treated using lateral condensation with BioRoot™ RCS. While these results are encouraging, they lack statistical weight due to sample size, and the condensation technique did not employ the same hydraulic pressure, potentially compromising homogeneity and sealer distribution [16].

Zavattini et al. documented 12-month healing rates of 90% (periapical radiographs) and 84% (CBCT) in a study involving 53 teeth obturated with BioRoot™ RCS via hydraulic condensation. The results of their study are comparable to ours evaluating the outcome of non-surgical endodontic treatments with BioRoot™ RCS using periapical radiographs for the assessment of the outcome of endodontic treatments. In addition, they used CBCT to assess the clinical outcome, representing an advantage in terms of accuracy when evaluating the outcome of root canal treatment in comparison with 2D radiographic approaches [6].

The current study assessed periapical healing over a 12-month period, providing insight into treatment success with hydraulic condensation and BioRoot™ RCS. While our results demonstrate a marked improvement in healing status, they align with the growing body of literature supporting the performance of calcium silicate-based sealers. Simon et al. (2025) and Hu et al. (2023) reported stable outcomes at two years, confirming sustained healing without relapse [20,21]. Similarly, Spinelli et al. (2024) and Bardini et al. (2025) extended this evidence base with three- and four-year follow-up data, indicating that the bioactivity and sealing integrity of bioceramic sealers are maintained in the long term [22,23]. These findings corroborate our results and support the potential of hydraulic condensation with BioRoot™ RCS as a reliable obturation protocol.

The findings of the present study expand upon previous in vivo investigations of tricalcium silicate-based sealers and hydraulic obturation like those of Bardini et al. (2021) and Zavattini et al. (2020), which have demonstrated the clinical viability of bioactive endodontic materials [6,17]. However, the distinctive contribution of the current work lies in the integration of these concepts within a strictly standardized clinical protocol. Every procedure—ranging from canal instrumentation and irrigation to sealer placement and hydraulic condensation—was conducted under controlled conditions, ensuring a high level of reproducibility. This allowed for a more precise assessment of the biological healing response, minimizing technique-related variability that may have influenced previous outcomes.

Cone-beam computed tomography is often cited as the superior imaging modality for assessing periapical changes due to its three-dimensional capabilities and sensitivity in detecting periapical bone defects. Nonetheless, the present clinical study adopted periapical radiographs for follow-up due to their routine use in daily practice, lower radiation dose, and accessibility. When standardized and interpreted using validated scoring systems such as the periapical index, two-dimensional radiography remains a reliable diagnostic tool for monitoring endodontic healing [28,29].

BioRoot™ RCS is a calcium silicate-based sealer with high biocompatibility, excellent sealing ability, and bioactive potential that fosters periapical healing. It releases calcium ions, stimulates mineralization, and demonstrates favorable interaction with periapical tissues [30,31,32]. These properties are enhanced when used with hydraulic condensation, a technique that ensures intimate contact between the sealer and dentin walls and minimizes voids [7,33]. Importantly, minimal extrusion was observed in this study a clinically significant finding, given the documented risks of delayed healing or pain associated with sealer extrusion [34,35].

A distinctive methodological element in this study was the drying protocol: canals were dried using a corresponding single paper point (from PTG system), without alcohol or over-drying by using additional paper point. This preserved the natural dentinal moisture necessary for the hydration and setting of the water-based BioRoot™ RCS, as confirmed by previous studies on calcium silicate sealers. Over-drying can compromise physical properties and bioactivity [8]. It is important to emphasize that the results of this study apply strictly to BioRoot™ RCS in its powder/liquid formulation. These findings should not be generalized to premixed or ready-to-use calcium silicate sealers, which may differ in terms of viscosity, setting behavior, ion release, and clinical handling properties [11,36,37].

Equally noteworthy is the one-visit approach. Although the role of interappointment calcium hydroxide has been historically debated, recent systematic review and meta-analyses report no significant advantage for multiple-visit endodontics, particularly when robust irrigation protocols are followed [34,38]. Our results support this, as all teeth, including those with a baseline PAI ≥ 3—achieved favorable outcomes within a single session. In our study, a rigorous irrigation protocol was employed, including the use of activated sodium hypochlorite and EDTA, followed by obturation with a tricalcium silicate-based sealer known for its bioactivity, sealing ability and antimicrobial properties, excluding the need of multi-visit treatment (no need of calcium hydroxide interappointment medication). We used 2.5% sodium hypochlorite (NaOCl) to balance antimicrobial efficacy with reduced cytotoxicity and risk of dentin erosion. Although 5.25% NaOCl offers higher tissue-dissolving capacity, previous studies have demonstrated that 2.5% provides adequate antimicrobial action while minimizing structural degradation and irritation to periapical tissues, which is particularly important in clinical settings [35]. This approach aligns with contemporary endodontics, increasingly supporting single-visit treatment in both irreversible pulpitis and apical periodontitis cases when selection and procedural execution are optimal. Moreover, single-visit treatment provides benefits from a patient-centered perspective, including reduced postoperative discomfort, fewer appointments, and improved compliance, all of which are important considerations in clinical practice. Nonetheless, we acknowledge that some clinicians may prefer a multi-visit approach, particularly in complex endodontic cases.

All treated teeth were restored with either composite resin or full coverage crowns (depending on residual structure) within one week of obturation. Coronal sealing plays a pivotal role in preventing reinfection, especially following root canal treatment, although some researchers have stated that the type of coronal restoration and the materials and procedure used do not affect the outcome of the treatment [39,40].

As a single-arm longitudinal observational study, the scientific rigor of our study was maintained through standardized treatment protocols, blinded PAI scoring, and within-subject comparisons of periapical healing (baseline vs. 6-/12-month follow-ups). This design allowed us to focus exclusively on the clinical behavior of this material without introducing variability from other obturation techniques. Given the strong evidence supporting the superior qualities of tricalcium silicate bioceramic endodontic sealers (BioRoot™ RCS), as well as the ethical considerations, it was not feasible to intentionally treat patients with inferior or outdated protocols solely for comparative purposes. Our study was therefore designed as a prospective cohort evaluation under clinical conditions, focusing on healing outcomes with this modern approach. Effect sizes and statistical significance were interpreted relative to literature data, with all subjects serving as their own controls [6,16,17]. The current study represents the evaluation of the clinical outcome of teeth obturated with hydraulic condensation and tricalcium silicate bioceramic sealer, at 6- and 12-month follow-up, showing no difference with other established contemporary approaches, such as zinc oxide-eugenol and resin based sealer and warm vertical compaction technique [6,17].

### 4.1. Limitations

This study has several limitations. Key limitation is the single-arm design without a concurrent control group (e.g., teeth treated with traditional techniques) limits direct comparative conclusions about treatment superiority. Second, while 12-month follow-up captures intermediate healing patterns, longer observation (24+ months) would better assess stability for chronic lesions. Third, standardization using a single experienced endodontist ensures procedural consistency but may introduce operator bias, limit generalizability and not reflect outcomes in general dental practice. Finally, radiographic assessment via periapical indices, though validated, lacks the sensitivity of CBCT for detecting early/minor anatomical changes. These factors were considered when interpreting results, suggesting need for future randomized trials with extended follow-up and multicenter operator variability.

A limitation of our protocol is that canal moisture was estimated clinically by the use of a single calibrated paper point per canal, without objective measurement. While this is in line with manufacturer recommendations and clinical practice, it introduces a degree of operator subjectivity. A further limitation of this study is the lack of subgroup stratification by diagnosis (irreversible pulpitis vs. apical periodontitis) and tooth type (single- vs. multi-rooted), which may influence treatment outcomes. Another limitation is the relatively high proportion of cases with baseline PAI-1 and PAI-2 scores, which may have positively influenced the overall healing outcomes and should be considered when interpreting the results.

Our clinical success reflects the specific characteristics and limitations of this study. First, our patient cohort was carefully selected, with strict inclusion and exclusion criteria applied to eliminate confounding factors such as open apices (immature teeth), extensive resorption, or severe periodontal disease. Second, the procedures were performed by professional endodontists under controlled clinical conditions, following standardized protocols for instrumentation, irrigation, obturation, and evaluation. Third, the follow-up period was limited to 12 months, during which some failures might not yet have manifested. Lastly, our definition for success, in line with contemporary clinical endodontics, included both complete healing and radiographic signs of ongoing improvement, without symptoms or loss of function. While we recognize that such a high success rate is uncommon in clinical settings, it may be attributed to the controlled nature of this prospective study, the 12-month follow-up, and the favorable clinical conditions, referring to the combination of asymptomatic status, functional integrity of the tooth, and absence of clinical signs of disease, suggesting that the healing process is progressing or has completed successfully. In the present study, we selected 6-month and 12-month follow-up intervals based on endodontic outcome studies and periapical healing timelines. The rationale of these timepoints is chosen as it represents a widely used benchmark for meaningful assessment of both short- and medium-term healing patterns, especially considering that the majority of radiographic healing changes occur within the first 6-12 months following treatment [20,37,40]. We fully acknowledge, however, that periapical healing, particularly in cases of severe apical periodontitis, may continue beyond 12 months. Future randomized trials comparing hydraulic condensation technique and tricalcium silicate sealers with traditional approaches, longer follow-up (e.g., 24–36 months), and inclusion of multiple operators would strengthen the external validity and confirm long-term clinical relevance.

### 4.2. Future Directions

Several directions for future research could be outlined based on the findings of this study. While the present investigation provides preliminary evidence supporting the clinical applicability of hydraulic condensation in combination with a tricalcium silicate–based sealer, further studies are warranted to validate and expand upon these results. Future randomized controlled clinical trials directly comparing the hydraulic condensation technique with other established obturation methods—such as warm vertical compaction or carrier-based obturation—using both resin-based and zinc oxide–eugenol sealers as reference groups would allow for more robust evaluation of relative clinical performance. Studies incorporating larger and more diverse patient cohorts, treated by multiple operators with varying levels of clinical experience, would provide insight into the generalizability and reproducibility of the technique in routine practice. Including multi-center designs could further minimize operator-specific and site-specific biases, thus increasing the external validity of the findings. Extending the follow-up period to at least 24–36 months would also be critical to assess the long-term stability of periapical healing and the durability of the obturation seal over time, as short- to medium-term follow-up (such as 12 months) cannot fully capture late failures or recurrence of apical pathology. In addition, incorporating standardized radiographic assessment criteria and, when feasible, advanced imaging modalities such as (CBCT) could provide more sensitive detection of periapical changes and enhance the reliability of outcome evaluation. Future research could benefit from incorporating patient-reported outcomes (e.g., postoperative pain, functional recovery, and overall satisfaction) to complement radiographic and clinical assessments. Such comprehensive outcome measures would contribute to a more patient-centered evaluation of endodontic success and support evidence-based decision-making in clinical practice.

## 5. Conclusions

This clinical study demonstrates that single-visit endodontic treatment using the hydraulic condensation technique with tricalcium silicate bioceramic sealer (BioRoot™ RCS) promotes predictable clinical healing outcomes. This approach encourages resolution of apical periodontitis in non-vital cases and supports the preservation of periapical health in teeth initially diagnosed with irreversible pulpitis. The significant improvement in radiographic PAI scores at 12 months confirms that this technique may serve as a viable method for endodontic treatment.

## Figures and Tables

**Table 1:** Pairwise comparisons (post hoc tests with Bonferroni correction). Significances is indicated with * for significant and ** for highly significant).

Comparison	Mean Difference (PAI)	*p*-Value
Baseline vs. 6-month	−1.36 ± 0.68	<0.001 **
6-month vs. 12-month	−0.39 ± 0.28	<0.003 *
Baseline vs. 12-month	−1.75 ± 0.82	<0.001 **

## Data Availability

The original contributions presented in the study are included in the article, further inquiries can be directed to the corresponding author.

## Abbreviations

The following abbreviations are used in this manuscript:

**Abbreviation**	**Definition**
CBCT	Cone-beam computed tomography
PAI	Periapical index
RCS	Root canal sealer
